# Selection of Suitable Reference Genes for Gene Expression Normalization Studies in *Dendrobium huoshanense*

**DOI:** 10.3390/genes13081486

**Published:** 2022-08-19

**Authors:** Shanyong Yi, Haibo Lu, Chuanjun Tian, Tao Xu, Cheng Song, Wei Wang, Peipei Wei, Fangli Gu, Dong Liu, Yongping Cai, Bangxing Han

**Affiliations:** 1 Department of Biological and Pharmaceutical Engineering, West Anhui University, Lu’an 237012, China; 2 Anhui Engineering Laboratory for Conservation and Sustainable Utilization of Traditional Chinese Medicine Resources, West Anhui University, Lu’an 237061, China; 3 College of Life Sciences, Anhui Agricultural University, Hefei 230036, China

**Keywords:** *Dendrobium huoshanense*, reference gene, qPCR, stability evaluation, abiotic stress

## Abstract

*Dendrobium huoshanense* is a kind of precious herb with important medicinal and edible value in China, which is widely used in traditional Chinese medicine for various diseases. Recent studies have paid close attention to the genetic expression of the biosynthetic pathway of the main active components (polysaccharides, alkaloids, and flavonoids), and real-time polymerase chain reaction (qPCR) is one of the most widely used methods for doing so. However, so far, no reference gene selections have been reported in *D. huoshanense*. In this study, 15 reference gene candidates (*GAPDH*, *eIF*, *EF-1α*, *PP2A*, *UBCE*, *RPL5*, *TBP*, *APT1*, *MDH*, *PTBP3*, *PEPC*, *CYP71*, *NCBP2*, *TIP41*, and *F-box*) were selected and evaluated for their expression stability in *D. huoshanense* under various experimental conditions, including in different tissues (root, stem, and leaf), abiotic stresses (oxidative, drought, cold, and UV), and hormone treatment (methyl jasmonate) using three statistical programs (geNorm, NormFinder, and BestKeeper). Then, the RefFinder program was employed to comprehensively validate the stability of the selected reference genes. Finally, the expression profiles of the *CESA* and *GMPP* genes were further analyzed, and these results indicated that *TBP*, *NCBP2*, and *CYP71* were the top three most stable reference genes after comprehensive comparison, which could be used as stable reference genes for normalizing the genes expression in *D. huoshanense.* This study described here provides the first data regarding on reference gene selection in *D. huoshanense*, which will be extremely beneficial for future research on the gene expression normalization in *D. huoshanense*.

## 1. Introduction

*D. huoshanense*, as named by C.Z. Tang and S.J. Cheng, is a perennial epiphytic herb listed in *Chinese Pharmacopoeia* as having high edible and medicinal value; it belongs to the *Dendrobium* genus of Orchidaceae [1]. The wild *D. huoshanense* has been in an extremely endangered state, caused by the low natural reproduction rate and long-term destructive picking [2]. However, it has been greatly protected and industrialized through tissue culture and artificial cultivation technology [3]. As one of the most important traditional Chinese medicines, it is also known as “soft gold”, and has been used as tonic for centuries [4,5]. Its stem is used as the medicinal part and it is generally considered that the plants with more juice and sticky teeth are the best [1]. For thousands of years, it was widely used as an ingredient in herbal medicines for nourishing yin and clearing heat, benefiting the stomach, and generating fluid [6]. The major active components of *D. huoshanense* are polysaccharides, flavonoids, sesquiterpenes, phenols, etc. [3,7,8,9]. Many pharmacological experiments showed that it has the effect of enhancing immunity, anti-oxidation, anti-aging, anti-tumor, and that it plays a role in reducing blood sugar, protecting the liver, etc. [10,11,12]. However, the research on the gene expression of *D. huoshanense* is still not deep enough. Some key genes involved in the biosynthetic pathways of important components are unclear or remain theorized [13,14,15,16,17]. Even though some studies have carried out quantitative research on gene expression, the use of internal reference genes is usually selected by experience. For example, in the quantitative studies of gene expression levels in *D. huoshanense* or related *Dendrobium* species, *Actin* [14], *Tubulin* [15], *18S rRNA* [16], *5.8S RNA* [18], *ASS*, and *APH1L* [19] were randomly used as reference genes for the normalization of candidate target genes, respectively.

In recent years, plenty of studies have carried out gene and protein research on the biosynthetic pathway of *D. huoshanense* [13,14,15,16,17,20,21]. With the development of next-generation sequencing (NGS) technology, it has become simpler and faster to analyze the distribution of mRNA and their expression levels in the biosynthetic pathway of this plant [22]. In addition, another analysis method, qPCR, is quietly popular in gene expression research, based on its high sensitivity, quantitative accuracy, throughput capability, and low cost using specific reference genes [23,24,25]. However, this quantitative result will naturally be disturbed by many factors, such as genome contamination, RNA quality, primer specificity, and amplification efficiency [26]. In order to guarantee accurate results and eliminate errors, one or more stable and acceptable reference genes are necessary.

Reference genes frequently come from common housekeeping genes, such as glyceraldehyde-3-phosphate dehydrogenase (*GAPDH*) [27], actin (*ACT*) [28], α-tubulin (*α-TUB*) [29], ribosomal RNA (*18S rRNA*) [30], and elongation factor 1α (*EF-1α*) [31]. They are usually used to calibrate qPCR results, so a stable reference gene is needed for qPCR. Nevertheless, several studies indicated that the expression levels of these traditional housekeeping genes varied widely in various experimental conditions among many species [32,33,34,35,36,37,38]. Consequently, selecting a stable and suitable reference gene has become increasingly crucial. However, there are no systematic studies for the selection of reference genes in *D. huoshanense* under external conditions (abiotic stress and hormone treatments). The research on the biosynthetic pathway mechanisms of the polysaccharides, alkaloids, and flavonoids of *D. huoshanense* is the main concern of researchers in this field. Although previous studies have described some candidate genes related to *D. huoshanense* [13,14,15,16,17,39], it is still necessary to find appropriate reference genes for further enhancing the detection of different gene expression levels of *D. huoshanense* under different experimental conditions by using qPCR.

Selecting out the stable reference genes of plant species is very important in gene expression research on the biosynthetic pathway of their main components [27,28,29,30,31,32,33,34,35,36,37,38]. In this study, based on the traditional housekeeping gene identity and the reports of plant stable internal reference genes in the literature [40,41,42], 15 candidate reference genes from *D. huoshanense*, namely *GAPDH*, *eIF*, *EF-1α*, *PP2A*, *UBCE*, *RPL5*, *TBP*, *APT1*, *MDH*, *PTBP3*, *PEPC*, *CYP71*, *NCBP2*, *TIP41*, and *F-box*, were assessed their expression stability by qPCR under abiotic stresses, namely oxidative (H_2_O_2_), drought (PEG), cold (4 °C), UV radiation (UV), and hormone treatment (methyl jasmonate (MeJA)), as well as in different tissues (root, stem, and leaf). Next, geNorm [43], NormFinder [44], and BestKeeper [45] were preliminarily employed to analyze the gene expression stability, and RefFinder (https://www.heartcure.com.au/reffinder/ (accessed on 1 August 2021)) was then used to comprehensively reassess the candidate genes. Finally, two genes related to polysaccharide biosynthesis, guanosine diphosphate (GDP)-mannose pyrophosphorylase (*GMPP*) [20] and cellulose synthase (*CESA*) [46], were selected to test and verify the stability of the selected internal reference genes. Our report of the selection of suitable reference genes for gene expression normalization studies in *D. huoshanense* continues to support that one or more stable and acceptable reference genes is very necessary to guarantee accurate results and eliminate errors. This study is the first systematic study of stable reference genes in *D. huoshanense*, which is helpful to promote the molecular research of *D. huoshanense*, especially in terms of gene expression involved in the biosynthesis of polysaccharides, alkaloids, flavonoids, etc.

## 2. Materials and Methods

### 2.1. Plant Materials

The plants used in the experiment were potted and planted in the greenhouse of the medicinal botanical garden of West Anhui University, which were identified as *D. huoshanense* by Professor Bangxing Han from West Anhui University. One-year-old *D. huoshanense* plants were used as experimental subjects. Specifically, the roots, stems and leaves of fresh *D. huoshanense* were used as samples of different tissues. For oxidative stress, the plant leaves were treated with 50 mM hydrogen peroxide (H_2_O_2_) once for 24 h. For drought treatment, 200 mL 25% PEG 6000 was used to treat plants every day for seven consecutive days. For temperature treatment, the plants were placed in a light incubator at 4 °C for two days. For UV treatment, the plants were exposed 15 cm from the UV light source (Philips TUV 30 W, 92 μW/cm^2^, 253 nm) for 15 min of radiation [47], and then a dark culture for 2 days. For hormone treatment, plant leaves were sprayed once with 25 mM MeJA and sampled after 6 h. All collected samples were quickly washed with distilled water and immediately frozen in liquid nitrogen and stored at −80 °C until RNA was extracted. Each group of samples had three biological replicates, and untreated *D. huoshanense* was used as control.

### 2.2. Total RNA Extraction and cDNA Synthesis

The total RNA of the cryopreservation samples from the refrigerator at −80 °C was extracted using an EASYspin Plus Complex Plant RNA kit (Aidlab, China), referring to the manufacturer’s instructions, and DNA was eliminated with DNase I (Aidlab, China). The purity and concentration of RNA samples were detected using 1% agarose gel electrophoresis and a Synergy H1 multifunction microplate detector (BioTek, American), respectively. Additionally, 0.2 μg of total RNA with a 260/280 ratio between 1.9 and 2.1 was used to synthesize first-strand cDNA using HiScript^®^ II Q RT SuperMix for qPCR (Vazyme, Nanjing, China) in accordance with the manufacture’s manual, and then the sample was diluted five times for qPCR analyses.

### 2.3. Selection of Candidate Reference Genes and Primers Design

Here, a total of 15 genes were selected as candidate reference genes by comparison with the TAIR database (http://www.arabidopsis.org (accessed on 7 February 2021)) using protein sequences of common housekeeping genes (*GAPDH*, *eIF*, *EF-1α*, *PP2A*, *UBCE*, *RPL5*, *TBP*, *APT1*, *MDH*, *PTBP3*, *PEPC*, *CYP71*, *NCBP2*, *TIP41*, and *F-box*) from *Arabidopsis thaliana* L. as templates. Then, the local BLAST program of BioEdit was employed to obtain the nucleotide sequences of putative *D. huoshanense* homologs based on the transcriptome data (access No. is PRJNA577972), and the information on 15 gene sequences of *D. huoshanense* was shown in Table S1. Next, Primer Premier 5 software was applied to design all the primers using the following principle: (1) the primer sequence length is 18–24 bp; (2) the amplification length is 80–300 bp; (3) the melting temperature (Tm) is 58–62 °C; (4) the GC content is 40–60%. Finally, all primers were synthesized by General Biol Company (Anhui, China) and checked by regular PCR products with 2% agarose gel electrophoresis before qPCR analysis. The melting curve was obtained to further determine primer specificity under the reaction condition at 95 °C for 15 s, 60 °C for 60 s, and 95 °C for 15 s using an AceQ qPCR SYBR Green Master Mix (Vazyme, Nanjing, China). The PCR efficiency (E) and correlation coefficient (R^2^) of each gene was obtained directly from the StepOne^TM^ Real-time PCR system (Applied Biosystems, Waltham, MA, USA) according to a standard curve generated from the 5-fold dilution cDNA series. All the information of the gene-specific primer pairs used in this study is listed in Table 1.

### 2.4. qPCR Analysis

According to the introduction of the AceQ qPCR SYBR Green Master Mix, the qPCR reaction system was conducted in a 20 µL mixture with 10 µL AceQ SYBR Green Master Mix (High ROX Premixed), 0.2 µM of forward and reverse primers, 2 µL diluted cDNA template mentioned in item 2.2, and 7.2 µL of RNase-free ddH_2_O. The reaction condition followed the illustration from the manufacturer, which is 95 °C for 5 min for pre-denaturation, 40 cycles of 95 °C for 10 s for denaturation, and 60 °C for 30 s for annealing/extension. All experiments were performed with three independent biological and technical repetitions.

### 2.5. Gene Expression Stability Analysis

In order to evaluate the stability of each candidate gene under various experimental conditions, including different tissues (root, stem, and leaf), abiotic stresses (oxidation, drought, cold, and UV) and hormone treatment (MeJA), three different algorithms, geNorm, NormFinder, and BestKeeper, were employed to carry out statistical analysis. Specifically, geNorm calculated the expression stability values (M) and pairwise variation comparison (Vn/Vn + 1). NormFinder assessed the reliability of reference genes based on their variations in both the intra-group and inter-group. Different to geNorm and NormFinder, which needed the 2^−∆Ct^ value [43,44], BestKeeper directly used the Ct values to rank the gene expression stability by calculating CV ± SD (coefficient of variation ± standard deviation) to choose the most stable genes.

### 2.6. Comprehensive Analysis and Validation of Selected Reference Genes

Firstly, the website tool RefFinder (https://www.heartcure.com.au/reffinder/ (accessed on 1 August 2021)) was used to comprehensively reassess the results of the candidate genes stability analysis from the geNorm, NormFinder, and BestKeeper softwares. Then, in order to examine the reliability of the selected reference genes, the two most stable and one least stable internal reference genes were used for normalizing the expression levels of two target genes (*GMPP* and *CESA*) [20,46] related to polysaccharide biosynthesis in different tissues and hormone treatment. Sample collections and experiments were conducted as mentioned above. The expression levels of two target genes were normalized by the 2^−∆∆Ct^ method [48].

## 3. Results

### 3.1. Primer Specificity Verification and PCR efficiency

**Figure 1:**
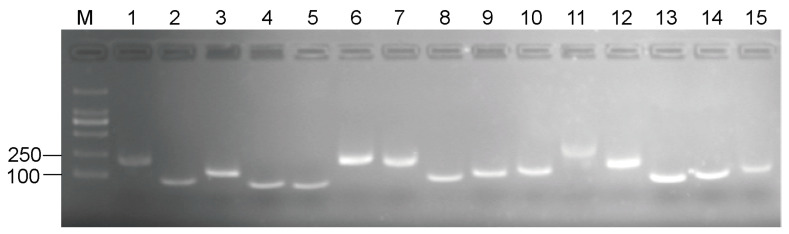
Specificity of primer pairs of all candidate reference genes for qPCR amplification. Here, M represents the DNA size marker. Lanes 1–15 are as follows: *TBP*, *UBCE*, *NCBP2*, *eIF*, *APT1*, *RPL5*, *GAPDH*, *EF-1α*, *F-box*, *TIP41*, *PEPC*, *PP2A*, *MDH*, *PTBP3*, and *CYP71*.

**Figure 2:**
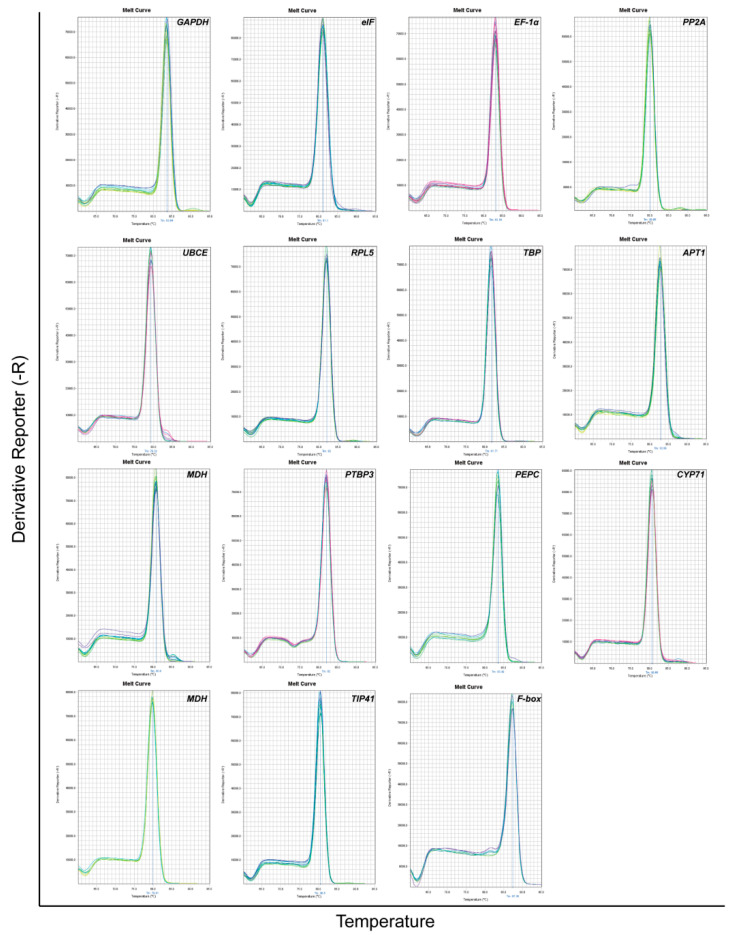
Specificity of primer pairs of qPCR amplification. Melting curves of 15 candidate reference genes exhibiting only single peaks.

To investigate the reference gene of *D. huoshanense*, 15 candidate genes (*GAPDH*, *eIF*, *EF-1α*, *PP2A*, *UBCE*, *RPL5*, *TBP*, *APT1*, *MDH*, *PTBP3*, *PEPC*, *CYP71*, *NCBP2*, *TIP41*, and *F-box*) were chosen based on previous studies [36,37] and the TAIR database. The specific amplification of primer pairs of all candidate reference genes was firstly checked by regular PCR, which exhibited a single PCR band meet the expected size in 2.0% agarose gel electrophoresis analysis (Figure 1). Moreover, the melting curve analysis showed a single amplification peak which further confirmed the specificity of the genes (Figure 2). According to the standard curve of each internal reference gene obtained by diluting cDNA in a 5-fold gradient, the amplification efficiency ranged from 90.502% for *PEPC* to 104.77% for *TBP*, and all the correlation coefficients (R^2^) were greater than 0.990 (Table 1).

### 3.2. Expression Profile of the Reference Genes

**Figure 3:**
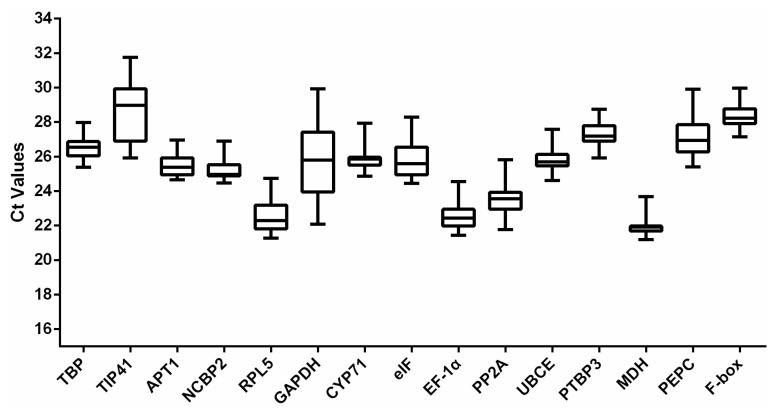
The comparison of raw cycle threshold (Ct) of the 15 candidate reference genes in different samples. The box graph indicates the 25th and 75th percentiles, with the lines in the center of the boxes indicate the medians. The whisker caps represent the maximum and minimum values, respectively.

The raw Ct values of the reference genes were collected and are exhibited in Figure 3. It can be observed that the number of cycles correspond to the threshold of fluorescence level which can be detected. The average Ct values of the 15 reference genes ranged from 22.00 to 28.58. According to the average Ct values, it can be judged that the expression abundance of 15 internal reference genes from high to low was *MDH* > *RPL5* > *EF-1α* > *PP2A* > *NCBP2* > *APT1* > *GAPDH* > *eIF* > *UBCE* > *CYP71* > *TBP* > *PEPC* > *PTBP3* > *F-box* > *TIP41*. Low Ct values correspond to high gene expression abundance, so the expression abundance of *MDH* was the highest and *TIP41* was the lowest. Here, *TBP*, *APT1*, *NCBP2*, and *MDH*, have relatively narrow Ct variation ranges, indicating that their expression levels may be more stable. However, considering the complexity and diversity of the plant growth environment, the stability of the selected internal reference genes still needs to be further investigated under different environmental conditions.

### 3.3. The Analysis of Expression Stability of Candidate Reference Genes

In order to improve the accuracy of analysis, three frequently used algorithms, namely geNorm, NormFinder, and BestKeeper, were separately employed to further assess the stability of candidate reference genes under different treatments and tissues.

#### 3.3.1. geNorm Analysis

**Figure 4:**
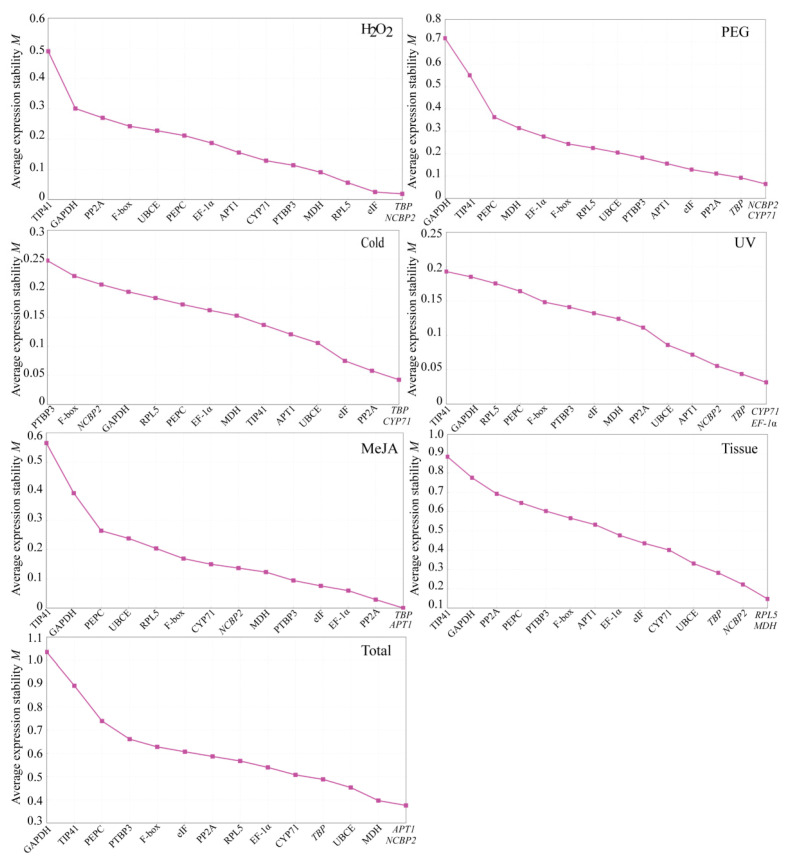
The rankings of the average expression stability values (M) of 15 reference genes using geNorm. Lower M values indicate more stable gene expression.

**Figure 5:**
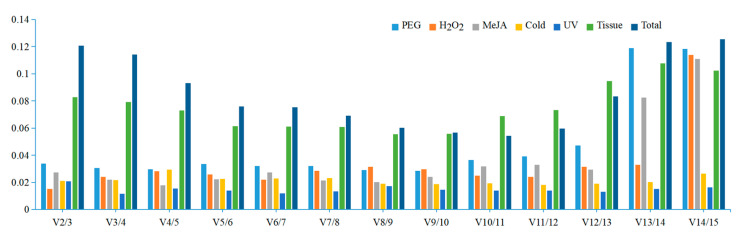
The pairwise variation values (Vn/Vn + 1) of all candidates calculated using the geNorm to confirm the optimal number of reference genes for qPCR data accurate normalization; the threshold used was 0.15, below which the inclusion of an additional reference gene is not necessary.

In geNorm analysis, measurement (M) values of expression stability of each reference gene is generated for each pair of genes. This ranks the expression stability by M values, and the lower M value represents the higher stability of gene expression. Generally, if M > 1.5, it could be regarded as an unsuitable reference gene. As shown in Figure 4, the stability trend of the 15 candidate internal reference genes in the tissue group, MeJA group, and different abiotic stress groups were arranged from high to low by the geNorm analysis, and the most stable internal reference genes given by all groups had some differences. Concretely, the five most stable internal reference genes in each group were as follows: *TBP* > *NCBP2* > *eIF* > *RPL5* > *MDH* for the H_2_O_2_ group; *NCBP2* > *CYP71* > *TBP* > *PP2A* > *eIF* for the PEG group; *TBP* > *CYP71* > *PP2A* > *eIF* > *UBCE* for the cold group; *CYP71* > *EF-1α* > *TBP* > *NCBP2* > *APT1* for the UV group; *TBP* > *APT1* > *PP2A* > *EF-1α* > *eIF* for the MeJA group; *RPL5* > *MDH* > *NCBP2* > *TBP* > *UBCE* for the tissue group; *APT1* > *NCBP2* > *MDH* > *UBCE* > *TBP* for the total group. More uniformly, for all samples, except for *PTBP3* and *F-box* in the cold group, the two most unstable genes in other groups were *TIP41* and *GAPDH*. In addition, geNorm can also recommend the required number of optimal internal reference genes according to the value of the pairwise variation (Vn/Vn + 1). When Vn/Vn + 1 is less than 0.15, the optimal number of reference genes is n [43]. According to this standard, the V2/V3 values are lower than 0.15 in all groups, so it is considered that the optimal number of internal reference genes is 2 (Figure 5), indicating that 2 reference genes can be sufficient for gene normalization.

#### 3.3.2. NormFinder Analysis

The NormFinder can directly evaluate the stability of reference genes, based on the variance in intra- and inter-group, to calculate the normalization factors using ANOVA. Generally, the lower stability value also reflects better stability of the corresponding reference gene expression. As shown in Table 2 from the top to the bottom, the stability values of reference genes are listed from the lowest to the highest with the NormFinder analysis. In detail, the most stable internal reference genes could be discovered clearly in all experimental conditions. The five most stable internal reference genes in each group were as follows: *TBP* > *NCBP2* > *eIF* > *RPL5* > *PTBP3* for the H_2_O_2_ group; *NCBP2 > CYP71 > TBP > PP2A > eIF* for the PEG group; *TBP > CYP71 > PP2A > APT1* > *UBCE* for the cold group; *EF-1α* *> CYP71 > NCBP2 > TBP* > *UBCE* for the UV group; *TBP* > *APT1* > *PP2A* > *EF-1α* > *eIF* for the MeJA group; *MDH > NCBP2 > RPL5 > UBCE* > *TBP* for the tissue group; *NCBP2* > *MDH* > *APT1* > *UBCE* > *TBP* for the total group. According to the comprehensive analysis, *TBP* can be regarded as a most stable reference gene, similar to the results with the geNorm analysis. In addition to the unstable expression of *NCBP2* in the cold and MeJA group, it was also very stable in other groups. Furthermore, the two most unstable genes (*GAPDH* and *TIP41*) in each group were consistent with the results of the geNorm analysis apart from the cold group. Combined with the analysis results of geNorm, we can judge the best combinations of the two internal reference genes in each group as follows: *TBP* + *NCBP2* for the H_2_O_2_ group; *NCBP2* + *CYP71* for the PEG group; *TBP* + *CYP71* for the cold group; *CYP71* + *EF-1α* for the UV group; *TBP* + *APT1* for the MeJA group; *RPL5* + *MDH* for the tissue group; *APT1* + *NCBP2* for the total group.

#### 3.3.3. BestKeeper Analysis

BestKeeper is an Excel-based program that directly uses the raw Ct values without conversion to calculate coefficient of variation (CV) and standard deviation (SD) of the reference gene from each experimental group, so as to compare their stability [45]. The smaller CV ± SD value, the more stable the reference gene. If the SD value is greater than 1, it is generally considered that this gene is unstable and should be discarded. All analysis results are shown in Table 3, and the stable arrangement of each gene is somewhat different from that of geNorm and NormFinder. Specifically, the five most stable internal reference genes in each group were as follows: *TBP* > *RPL5* > *NCBP2* > *eIF* > *PTBP3* for the H_2_O_2_ group; *NCBP2* > *CYP71* > *TBP* > *eIF* > *PP2A* for the PEG group; *EF-1α* > *UBCE* > *CYP71* > *APT1* > *TBP* for the cold group; *EF-1α* > *CYP71* > *TBP* > *UBCE* > *NCBP2* for the UV group; *eIF* > *EF-1α* > *TBP* > *APT1* > *NCBP2* for the MeJA group; *PTBP3* > *PEPC* > *UBCE* > *TBP* > *EF-1α* for the tissue group; *MDH* > *TBP* > *PTBP3* > *UBCE* > *CYP71* for the total group. However, for most treatments, *TBP* or *NCBP2* still showed good stability, basically consistent with the geNorm and NormFinder analyses. In addition, except for the Cold group, *GAPDH* and *TIP41* were still more unstable than others, a result which was nearly close to the results of geNorm and NormFinder analysis.

### 3.4. Comprehensive Analysis and Validation of Reference Genes

Considering the differences in stability analysis from the first three programs, we further used the online comprehensive ranking platform RefFinder, which used geNorm, Normfinder, BestKeeper, and the comparative ΔCt method to verify the rankings of candidate reference genes [49] (Table 4). The geometric mean of the attributed weight from the stable value of each gene was calculated by RefFinder, and the smaller the value, the more stable the internal parameter gene. The ranking order of the top five most stable and unstable candidate reference genes acquired by RefFinder were basically the same as the results provided by geNorm and Normfinder, which is slightly different from the results of BestKeeper. For example, in all treatment groups, the first two most unstable genes generated by RefFinder were basically the same as the first three most unstable genes calculated by geNorm, Normfinder, and BestKeeper. Moreover, the stability ranking order of the 15 reference genes was counted in Table S2 to better observe the rankings of the 4 software analysis results, which suggested that *TBP*, *NCBP2*, and *CYP71* could be the 3 best stable genes in *D. huoshanense* under the different conditions.

**Figure 6:**
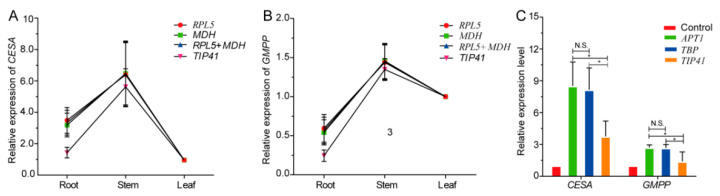
Relative expression levels of *CESA* and *GMPP* normalized by the selected reference genes in different tissues (root, stem, and leaf) and methyl jasmonate (MeJA) treatment. (**A**) The *CESA* expression level on different tissues; (**B**) The *GMPP* expression level in different tissues; (**C**) The *CESA* and *GMPP* expression level in the stem under MeJA treatment (* *p* < 0.05; Here, N.S. indicates no significant difference). The error bars represent the mean ± SD of three biological replicates.

Then, in order to further verify the reliability of the screened stable internal reference genes, the expression of two genes (*CESA* and *GMPP*) related to polysaccharide biosynthesis pathway in different tissues (root, stem, and leaf) and MeJA treatment for stem were analyzed by qPCR with the unstable reference gene (*TIP41*), three stable reference genes (*RPL5, MDH,* and *TBP*), and their combinations as reference genes based on the comprehensive rankings mentioned in Table S2. The results showed that, when *RPL5*, *MDH* and *RPL5* + *MDH* were used as internal reference genes, the expression levels of *CESA* and *GMPP* were slightly different in the root, stem, and leaf samples. Among them, the expression levels of *CESA* were stem > root > leaf, and the expression levels of *GMPP* were stem > leaf > root (Figure 6A). However, when the unstable internal reference gene *TIP41* was used, the relative expression levels of *CESA* and *GMPP* in root and stem were significantly different from the quantitative results of *RPL5*, *MDH,* and *RPL5* + *MDH* (Figure 6B). Likewise, when the stable *APT1* or *TBP* was selected as internal reference gene, it was obvious that the relative quantitative results of *CESA* or *GMPP* in the stem were similar, and while using the unstable internal reference gene *TIP41*, the relative quantitative results of *CESA* or *GMPP* in stem were significantly different (Figure 6C).

## 4. Discussion

*D. huoshanense* has attracted many researchers because of its broad pharmacological activity [9]. Our research group on *D. huoshanense* has carried out a series of studies on the field resource protection, tissue culture and cultivation, chemical component separation and identification, bioactivity analysis and action mechanism, and new drug and product development [3,5,10,39,50,51,52,53], and also completed its whole genome sequencing [54]. In addition, the transcriptome sequencing of *D. huoshanense* had been reported several times in other research groups [13,14,15,16,17]. Since the main active components of similar plants have been gradually known, many studies also mainly focused on exploring and understanding the biological control [55] and biosynthetic pathways of polysaccharides, alkaloids and flavonoids, etc. [13,14,15,16,17].

The gene expression level has an inevitable and important role in the biosynthesis of secondary metabolites and related gene function mining. Due to its high sensitivity and specificity, qPCR is often used for high-throughput analysis at the level of gene transcription. To obtain accurate and reliable results for the data, a stable and suitable reference gene is required. In fact, in the quantitative studies of the genetic expression of *D. huoshanense* or its related species, multiple genes were randomly used as internal reference genes [14,15,16,17,18,19], which would cause errors in the analysis results. To our knowledge, there are still no systematical studies on reference genes in *D. huoshanense.* Moreover, there are three main cultivation methods of *D. huoshanense* including facility cultivation, under forest cultivation, and simulative habitat cultivation [3]. Among these, the simulative habitat cultivation of *D. huoshanense* has the comprehensive effects of “excellent environment”, “excellent shape”, “high quality”, and “excellent effect” [3], which is similar to the report of *Dendrobium officinale* [56]. This cultivation method has almost no manual intervention in the growth process, and is mainly faced with various environmental stress factors, such as UV, temperature, and drought. In order to further study the molecular mechanism of the effects of environmental factors on the quality of *D. huoshanense*, it is necessary to screen the internal reference genes that can be stably expressed in different tissues, and under the main abiotic stress conditions (oxidation, drought, temperature, and ultraviolet radiation) and hormone stress. Thus, we set up six treatments in this study, and the results also confirmed the necessity of this configuration. Therefore, we comprehensively analyzed the expression levels and stability of 15 candidate reference genes under corresponding conditions.

All raw Ct values of *D. huoshanense* samples under different abiotic conditions, hormone treatment, and in different tissues were acquired from qPCR and processed using geNorm, NormFinder, and BestKeeper for ranking the reference genes. According to the results of this study, the 15 candidate reference genes firstly exhibited a relatively reliable range for expression profiles from 22.00 to 28.58, revealing that the selected candidate genes were able to offer an accurate normalization. Based on the relatively narrow Ct variation ranges, *TBP*, *APT1*, *NCBP2*, and *MDH* might be tentatively considered as the better stable reference genes (Figure 3). Obviously, this results on the rankings of 15 candidate reference genes were somewhat different from the outcomes calculated by geNorm, NormFinder, and BestKeeper, which indicated that it is necessary to use multiple procedures to obtain the best results (Figure 4, Table 2 and Table 3), and also revealed that none of the 15 reference genes could be stably expressed for meeting all conditions in *D. huoshanense*. Next, three Excel-based programs were further employed to estimate the stability of the candidate genes, and their results showed some differences in ranking order because their analysis principle and emphasis are different. In the analysis of geNorm and NormFinder, the five most stable genes given by them were basically the same, while the difference was their different stability ranking order. However, the analysis results of BestKeeper are different from those of geNorm and NormFinder, mainly due to the CV and SD values, which are the key factors for determining the stability ranking of reference genes obtained by BestKeeper. This discrepancy was acceptable because the results of the three methods showed some consistency in selecting the five most stable genes. Eventually, the above three analysis results were synthesized by RefFinder, and the five most stable genes were close to those given by geNorm and NormFinder. Combining the results of these four software analysis (Table S2), the five most stable genes were as follows: *TBP* > *NCBP2* > *RPL5* > *eIF* > *PTBP3* in the H_2_O_2_ group; *NCBP2* > *CYP71* > *TBP* > *PP2A* > *eIF* in the PEG group; *TBP* > *CYP71* > *PP2A* > *UBCE* > *APT1* in the cold group; *EF-1α* > *CYP71* > *TBP* > *NCBP2* > *UBCE* in the UV group; *TBP* > *APT1* > *eIF* > *EF-1α* > *PP2A* in MeJA group; *TBP* > *UBCE* > *MDH* > *RPL5* > *NCBP2* in the tissue group; *MDH* > *TBP* > *UBCE* > *NCBP2* > *APT1* in the total group. Accordingly, these results demonstrated that *TBP*, *NCBP2*, and *CYP71* could be comprehensively regarded as the top three stable reference genes in *D. huoshanense*. Unexpectedly, although *GAPDH* is a commonly used reference gene, its expression in *D. huoshanense* showed fairly poor expression stability under all experimental conditions in our research, which meant that it could not be used as a stable reference gene for qPCR analysis. However, it was regarded as the best reference gene in *D. catenatum* [57], *Diabrotica undecimpunctata* [58], and *Corydalis yanhusuo* [59]. Similarly, although *TIP41* is not suitable as an internal reference gene in this study, its expression presented high stability in *Momordica charantia* [60]. These results further illustrated that there is no universally applicable reference gene with invariant expression, and that the selection of stable reference genes remain indispensable. Furthermore, in qPCR analysis, multiple reference genes are better than a single reference gene for normalization. According to the pairwise variation results (Figure 5), the combination of two reference genes should be enough to meet the demand for the normalization in different tissues and under all the experimental conditions (Figure 6).

In order to further confirm the suitability of the selected reference genes, two pairs of the most stable genes (*RPL5* and *MDH*, and *APT1* and *TBP*) and their combinations and one least stable gene (*TIP41*) were selected for the normalization of *CESA* [46] and *GMPP* [20] involved in the polysaccharide biosynthesis of *D. huoshanense* under MeJA treatments and in various tissues. Noticeably, when the selected stable reference gene pairs or their combinations were standardized alone, there were only slight differences in the relative expression levels of *CESA* and *GMPP* between different tissues and MeJA treatment. However, when using the least stable reference gene, a significantly different result was found in the relative expression levels of *CESA* and *GMPP* between different tissues and MeJA treatments, indicating that the selection and confirmation of appropriate and stable reference genes is particularly critical before they are used for a set of samples.

## 5. Conclusions

To the best of our knowledge, our study is the first to systematically explore and evaluate the expression stability of 15 reference genes from *D. huoshanense* under different abiotic stress factors, hormone treatment, and in different tissues. The results indicated that *TBP*, *NCBP2*, and *CYP71* could be regarded as the optimal reference genes based on their better stability in different conditions when analyzed by four commonly used programs (geNorm, NormFinder, BestKeeper, and RefFinder). Furthermore, the two unstable genes of each group were basically identical according to the comprehensive ranking order from the four programs at their relevant conditions. Finally, the validation experiments on the expression analysis of *CESA* and *GMPP* further accentuated that it is necessary to screen stable reference genes for the normalization of gene expression analysis by qPCR under different conditions. This study will be useful to increase the accuracy of gene expression analysis by qPCR and promote future research on gene functions in *D. huoshanense* and related *Dendrobium* species. Therefore, this research should also arouse great interest in researchers engaged in the mining of key genes in plant secondary metabolic pathways, inspiring further effort in plant molecular research and natural product biosynthesis pathway analysis.

## Figures and Tables

**Table 1:** Candidate genes and primer pairs used for qPCR normalization in *D. huoshanense*.

Gene Symbol	Gene Name	Arabidopsis Homolog Locus	Primer Sequence (5′–3′)	Amplicon Length (bp *)	Primers Tm * (°C)	E * (%)	R^2^ *
*GAPDH*	Glyceraldehyde-3-phosphate dehydrogenase	AT1G16300.1	F: TGCTTACGGCGATTACTTCC	279	58.2/58.6	96.979	0.994
			R: CTTCCAATACGACCAAAACCAT				
*eIF*	Eukaryotic translation initiation factor	AT3G60240.2	F: CCATTCCATCTGTCCCCCC	121	61.8/60.1	97.101	0.998
			R: GGACCCCTAAACGGAAAACATA				
*EF-1α*	Elongation factor 1-α-like	AT1G07920.1	F: GCCCAACCGATAAGCCACT	138	59.8/59.1	92.403	0.997
			R: TGAGTCCAGTAGGTCCGAAGG				
*PP2A*	Serine/threonine protein phosphatase 2A	AT3G2580.1	F: CGCTGCTCTTGGAAAACTGT	210	58.0/59.0	96.771	0.998
			R: ACTTCTGCCTCATTATCACGGA				
*UBCE*	Ubiquitin conjugating enzyme E2	AT1G50490.1	F: AATAGATGGAGGCAAGGGAACT	123	59.0/59.0	102.92	0.997
			R: TTGGGATGGAAACAGGAGGT				
*RPL5*	Ribosomal protein L5	AT5G39740.1	F: CGATTTGTTGTGCGATTTACG	270	59.5/59.4	97.704	0.996
			R: CCTCCTGCTTTCTGCTGGTT				
*TBP*	TATA box binding protein like	AT1G55520.1	F: GGCATCCTTCTGGTATTGTCC	252	58.5/58.4	104.77	0.998
			R: TACGAGCATACTTCCGAGCAG				
*APT1*	Adenine phosphoribosyltransferase 1	AT1G27450.1	F: ATGGCGTCCGTGGATGAA	114	59.8/58.8	93.636	0.999
			R: CAGCAGCAGCGTCGTGATA				
*MDH*	Malate dehydrogenase	AT5G43330.1	F: TTGCTGATGATGAGTGGCTGAG	142	61.0/59.5	98.403	0.991
			R: CCAAGGACCCAATCACGAAT				
*PTBP3*	Polypyrimidine tract binding protein homolog 3	AT1G43190.1	F: CACCTGACACCCGTGAGTTTG	209	60.9/61.2	93.938	0.998
			R: TTGCCTCTTACCATTCACCTCTATC				
*PEPC*	Phosphoenolpyruvate carboxykinase	AT4G37870.1	F: TGACATCATCCACAAGCATAGACA	283	60.5/59.4	90.502	0.995
			R: GAAACACCATTATCACTCCAGCA				
*CYP71*	Cyclophilin 71	AT3G44600.1	F: TGAATGGGTCTACAAACAAGGAG	227	58.7/59.6	92.806	0.993
			R: GCAATGTTGTAGGGCTCCAGTA				
*NCBP2*	nuclear cap binding protein subunit 2	AT5G44200.1	F: GACTCCCTGTGGCTTTTGCT	146	59.2/59.4	100.38	0.999
			R: GACCCCATTGTCTTCCTTCTTC				
*TIP41*	TIP41-like protein	AT4G34270.1	F: TGGCAGCGAAGCAGTAGAAC	200	60.2/60.8	101.45	0.996
			R: GAACTTTTACGGTCAAGAGGGAT				
*F-box*	F-box protein	AT5G39450.1	F: TTTCCCCGCAGTTTTCACG	169	58.9/58.8	102.65	0.990
			R: TTGGAACCTTCAGGCGGACT				
*GMPP*	GDP-mannose pyrophosphorylase	AT1G74910.1	F: GAATGTTCCGAAGCCGTTGT	130	58.9/58.6	94.465	1
			R: AGCAAACTCCCGTTCCTCAT				
*CESA*	cellulose synthase A	AT3G03050.1	F: CTTTGTTTCAACTGCTGACCCT	193	58.8/58.2	94.540	0.999
			R: ACGACAGAAAGGAACCCATAGA				

* Here, bp, Tm, E, and R^2^ mean base pair, melting temperature, PCR efficiency, and correlation coefficient, respectively. Standard curves of 15 candidate reference genes and two target genes were shown in Figure S1.

**Table 2:** Expression stability rank of 15 candidate reference genes by NormFinder.

Rank	H_2_O_2_	PEG	Cold	UV	MeJA	Tissue	Total
**1**	*TBP*(0.006)	*NCBP2*(0.022)	*TBP*(0.013)	*EF-1α*(0.012)	*TBP*(0.015)	*MDH*(0.145)	*NCBP2*(0.143)
**2**	*NCBP2*(0.006)	*CYP71*(0.022)	*CYP71*(0.029)	*CYP71*(0.023)	*APT1*(0.015)	*NCBP2*(0.147)	*MDH*(0.205)
**3**	*eIF*(0.009)	*TBP*(0.029)	*PP2A*(0.043)	*NCBP2*(0.037)	*PP2A*(0.015)	*RPL5*(0.162)	*APT1*(0.258)
**4**	*RPL5*(0.027)	*PP2A*(0.029)	*APT1*(0.055)	*TBP*(0.046)	*EF-1α*(0.027)	*UBCE*(0.236)	*UBCE*(0.280)
**5**	*PTBP3*(0.038)	*eIF*(0.033)	*UBCE*(0.068)	*UBCE*(0.050)	*eIF*(0.032)	*TBP*(0.283)	*TBP*(0.293)
**6**	*MDH*(0.047)	*UBCE*(0.067)	*eIF*(0.089)	*APT1*(0.052)	*PTBP3*(0.041)	*CYP71*(0.289)	*CYP71*(0.307)
**7**	*CYP71*(0.091)	*APT1*(0.099)	*TIP41*(0.114)	*PTBP3*(0.089)	*MDH*(0.070)	*F-box*(0.336)	*PP2A*(0.349)
**8**	*APT1*(0.175)	*PTBP3*(0.130)	*EF-1α*(0.114)	*F-box*(0.119)	*NCBP2*(0.089)	*APT1*(0.336)	*EF-1α*(0.355)
**9**	*EF-1α*(0.216)	*RPL5*(0.190)	*GAPDH*(0.126)	*PEPC*(0.122)	*CYP71*(0.144)	*eIF*(0.339)	*RPL5*(0.381)
**10**	*UBCE*(0.218)	*MDH*(0.231)	*PEPC*(0.129)	*MDH*(0.127)	*F-box*(0.194)	*PTBP3*(0.440)	*eIF*(0.381)
**11**	*F-box*(0.238)	*F-box*(0.239)	*MDH*(0.131)	*RPL5*(0.130)	*RPL5*(0.235)	*EF-1α*(0.447)	*F-box*(0.394)
**12**	*PEPC*(0.250)	*EF-1α*(0.310)	*NCBP2*(0.163)	*PP2A*(0.131)	*PEPC*(0.238)	*PEPC*(0.575)	*PTBP3*(0.507)
**13**	*PP2A*(0.264)	*PEPC*(0.387)	*RPL5*(0.170)	*eIF*(0.142)	*UBCE*(0.262)	*PP2A*(0.593)	*PEPC*(0.724)
**14**	*GAPDH*(0.300)	*TIP41*(1.214)	*F-box*(0.202)	*TIP41*(0.146)	*GAPDH*(0.844)	*GAPDH*(0.803)	*TIP41*(1.22)
**15**	*TIP41*(1.18)	*GAPDH*(1.23)	*PTBP3*(0.274)	*GAPDH*(0.149)	*TIP41*(1.15)	*TIP41*(1.04)	*GAPDH*(1.31)

**Table 3:** Expression stability values (CV ± SD) of internal reference genes calculated by BestKeeper.

Rank	H_2_O_2_	PEG	Cold	UV	MeJA	Tissue	Total
1	*TBP*	*NCBP2*	*EF-1α*	*EF-1α*	*eIF*	*PTBP3*	*MDH*
	0.13 ± 0.03	0.10 ± 0.02	0.47 ± 0.10	0.21 ± 0.05	0.37 ± 0.09	1.42 ± 0.39	1.62 ± 0.36
2	*RPL5*	*CYP71*	*UBCE*	*CYP71*	*EF-1α*	*PEPC*	*TBP*
	0.15 ± 0.03	0.20 ± 0.05	0.53 ± 0.14	0.34 ± 0.08	0.40 ± 0.09	2.02 ± 0.56	1.65 ± 0.44
3	*NCBP2*	*TBP*	*CYP71*	*TBP*	*TBP*	*UBCE*	*PTBP3*
	0.17 ± 0.04	0.21 ± 0.06	0.53 ± 0.14	0.36 ± 0.10	0.55 ± 0.14	2.06 ± 0.54	1.73 ± 0.47
4	*eIF*	*eIF*	*APT1*	*UBCE*	*APT1*	*TBP*	*UBCE*
	0.21 ± 0.05	0.40 ± 0.11	0.59 ± 0.15	0.40 ± 0.09	0.57 ± 0.14	2.09 ± 0.56	1.90 ± 0.49
5	*PTBP3*	*PP2A*	*TBP*	*NCBP2*	*NCBP2*	*EF-1α*	*CYP71*
	0.39 ± 0.10	0.47 ± 0.11	0.77 ± 0.20	0.42 ± 0.10	0.62 ± 0.15	2.11 ± 0.49	1.92 ± 0.50
6	*MDH*	*APT1*	*NCBP2*	*PTBP3*	*MDH*	*F-box*	*NCBP2*
	0.59 ± 0.13	0.62 ± 0.16	0.82 ± 0.21	0.42 ± 0.12	0.64 ± 0.14	2.32 ± 0.66	2.03 ± 0.51
7	*CYP71*	*UBCE*	*GAPDH*	*MDH*	*PTBP3*	*NCBP2*	*F-box*
	0.60 ± 0.15	0.67 ± 0.17	0.84 ± 0.21	0.44 ± 0.11	0.64 ± 0.17	2.69 ± 0.69	2.18 ± 0.62
8	*APT1*	*PTBP3*	*PTBP3*	*PEPC*	*PP2A*	*CYP71*	*APT1*
	0.94 ± 0.24	0.73 ± 0.20	0.84 ± 0.22	0.45 ± 0.11	0.67 ± 0.15	2.96 ± 0.78	2.27 ± 0.58
9	*UBCE*	*RPL5*	*PP2A*	*APT1*	*CYP71*	*APT1*	*EF-1α*
	0.97 ± 0.25	0.97 ± 0.23	0.91 ± 0.21	0.45 ± 0.12	0.85 ± 0.21	3.09 ± 0.80	2.68 ± 0.61
10	*F-box*	*F-box*	*TIP41*	*RPL5*	*F-box*	*MDH*	*eIF*
	0.98 ± 0.27	1.06 ± 0.30	0.93 ± 0.28	0.52 ± 0.11	0.94 ± 0.26	3.12 ± 0.70	2.76 ± 0.71
11	*PEPC*	*MDH*	*RPL5*	*PP2A*	*PEPC*	*RPL5*	*PP2A*
	1.06 ± 0.29	1.54 ± 0.34	0.98 ± 0.21	0.52 ± 0.14	1.21 ± 0.32	3.27 ± 0.76	2.84 ± 0.67
12	*EF-1α*	*EF-1α*	*MDH*	*F-box*	*UBCE*	*eIF*	*PEPC*
	1.15 ± 0.25	1.77 ± 0.40	1.06 ± 0.23	0.57 ± 0.16	1.47 ± 0.39	3.34 ± 0.88	3.12 ± 0.84
13	*GAPDH*	*PEPC*	*eIF*	*eIF*	*RPL5*	*GAPDH*	*RPL5*
	1.19 ± 0.32	1.81 ± 0.48	1.09 ± 0.27	1.05 ± 0.26	1.61 ± 0.35	3.92 ± 1.09	3.27 ± 0.74
14	*PP2A*	*TIP41*	*F-box*	*TIP41*	*GAPDH*	*TIP41*	*TIP41*
	1.45 ± 0.34	5.01 ± 1.40	1.27 ± 0.36	1.10 ± 0.25	3.89 ± 0.99	4.97 ± 1.40	5.40 ± 1.54
15	*TIP41*	*GAPDH*	*PEPC*	*GAPDH*	*TIP41*	*PP2A*	*GAPDH*
	5.01 ± 1.44	5.49 ± 1.45	1.96 ± 0.28	1.13 ± 0.25	4.72 ± 1.31	5.28 ± 1.26	6.99 ± 1.81

**Table 4:** Expression stability of candidate internal reference genes by RefFinder.

Rank	H_2_O_2_	PEG	Cold	UV	MeJA	Tissue	Total
**1**	*TBP*(1.41)	*CYP71*(1.41)	*TBP*(1.97)	*EF-1α*(1.63)	*APT1*(1.41)	*MDH*(1.68)	*MDH*(1.41)
**2**	*RPL5*(2.00)	*NCBP2*(1.57)	*CYP71*(2.45)	*CYP71*(2.45)	*TBP*(2.34)	*RPL5*(2.71)	*NCBP2*(1.57)
**3**	*NCBP2*(2.21)	*TBP*(2.45)	*PP2A*(2.99)	*NCBP2*(3.83)	*EF-1α*(2.63)	*NCBP2*(3.35)	*UBCE*(3.72)
**4**	*eIF*(3.22)	*PP2A*(3.94)	*UBCE*(3.13)	*APT1*(3.98)	*PP2A*(3.46)	*UBCE*(3.94)	*TBP*(3.76)
**5**	*PTBP3*(5.00)	*eIF*(4.73)	*APT1*(5.05)	*TBP*(4.79)	*eIF*(3.98)	*TBP*(4.47)	*CYP71*(4.82)
**6**	*MDH*(6.24)	*APT1*(6.70)	*EF-1α*(5.48)	*PEPC*(5.42)	*MDH*(5.66)	*PTBP3*(5.90)	*APT1*(5.01)
**7**	*CYP71*(6.74)	*UBCE*(6.70)	*eIF*(6.24)	*UBCE*(6.45)	*PTBP3*(6.45)	*EF-1α*(6.48)	*PP2A*(7.65)
**8**	*APT1*(8.24)	*PTBP3*(7.48)	*GAPDH*(7.65)	*PTBP3*(8.37)	*NCBP2*(7.74)	*CYP71*(6.82)	*EF-1α*(8.00)
**9**	*EF-1α*(8.97)	*RPL5*(9.00)	*TIP41*(8.57)	*RPL5*(8.66)	*CYP71*(9.00)	*F-box*(7.61)	*PTBP3*(8.49)
**10**	*UBCE*(9.97)	*F-box*(10.2)	*MDH*(9.06)	*MDH*(9.84)	*F-box*(10.0)	*eIF*(8.53)	*RPL5*(9.67)
**11**	*PEPC*(11.2)	*MDH*(11.5)	*NCBP2*(9.16)	*F-box*(10.1)	*RPL5*(11.2)	*APT1*(9.19)	*eIF*(10.2)
**12**	*F-box*(11.5)	*EF-1α*(11.7)	*PEPC*(11.5)	*PP2A*(10.4)	*PEPC*(12.2)	*PEPC*(9.64)	*F-box*(10.5)
**13**	*PP2A*(13.2)	*PEPC*(13.0)	*RPL5*(12.5)	*TIP41*(10.6)	*UBCE*(12.5)	*PP2A*(13.2)	*PEPC*(13.0)
**14**	*GAPDH*(13.7)	*TIP41*(14.0)	*PTBP3*(12.7)	*GAPDH*(11.0)	*GAPDH*(14.0)	*GAPDH*(13.7)	*TIP41*(14.0)
**15**	*TIP41*(15.0)	*GAPDH*(15.0)	*F-box*(14.2)	*eIF*(12.3)	*TIP41*(15.0)	*TIP41*(15.0)	*GAPDH*(15.0)

## Data Availability

The data presented in this study are openly available in GeneBank.

## Supplementary Materials

The following are available online at https://www.mdpi.com/article/10.3390/genes13081486/s1, Table S1: Information of 15 candidate reference genes and two target genes selected for evaluation and validation in *D. huoshanense*. Table S2: Expression stability rankings of the 15 candidate reference genes estimated by geNorm, NormFinder, BestKeeper and RefFinder. Figure S1: Standard curves of 15 candidate reference genes and two target genes were directly generated by StepOne^TM^ Real-time PCR system.
