# Supplementary material for: Selection of Suitable Reference Genes for Gene Expression Normalization Studies in Dendrobium huoshanense
Source: Genes (Basel). 2022 Aug 19;13(8):1486. doi: 10.3390/genes13081486 (PMC9408602; doi:10.3390/genes13081486)
Supplement: Supplementary file 1 [file genes-13-01486-s001.zip › Figure S1.pdf]

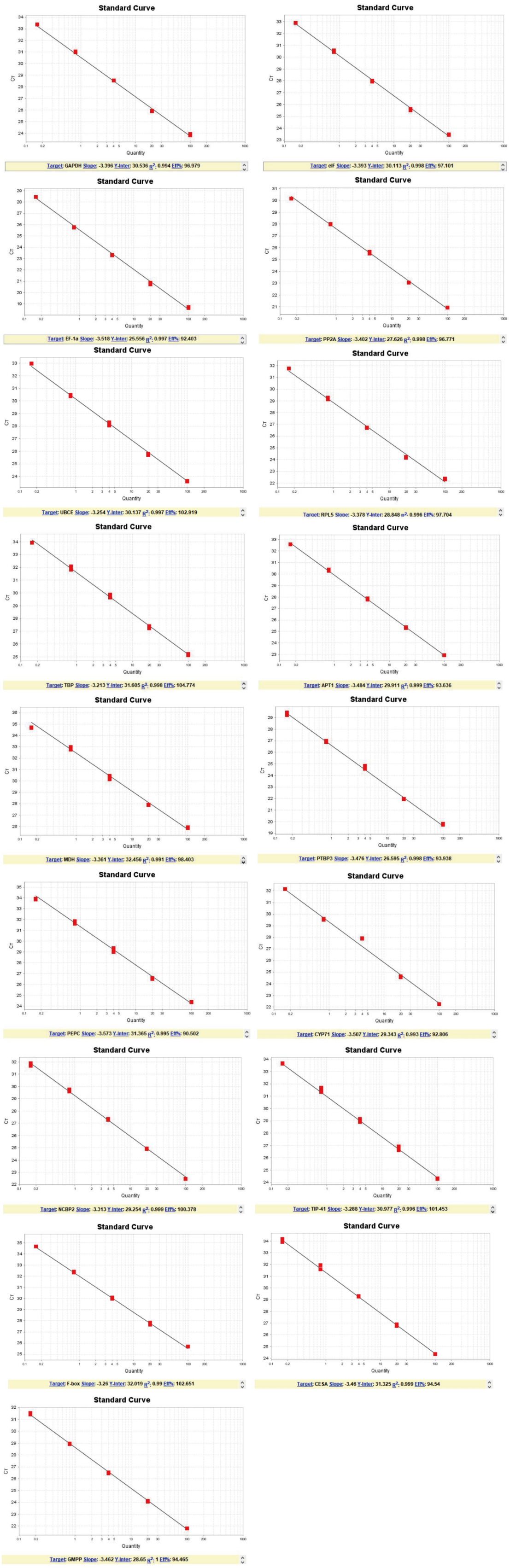

Figure S1. Standard curves of 15 candidate reference genes and two target genes were directly generated by StepOne^{TM} Real-time PCR system.
